# Diabetes mellitus and urinary tract infection: Causative uropathogens, their antibiotic susceptibility pattern and the effects of glycemic status

**DOI:** 10.12669/pjms.36.7.2881

**Published:** 2020

**Authors:** Shahzad Ahmad, Arshad Hussain, Mohammad Sajjad Ali Khan, Najmush Shakireen, Iftikhar Ali

**Affiliations:** 1 Dr. Shahzad Ahmad, MBBS, FCPS (Medicine), PGT (Endo). Department of Medicine & Allied, Northwest General Hospital & Research Centre, Peshawar, Khyber Pakhtunkhwa, Pakistan; 2 Dr. Arshad Hussain, FRCP (Edinburgh). Department of Medicine & Allied, Northwest General Hospital & Research Centre, Peshawar, Khyber Pakhtunkhwa, Pakistan; 3 Dr. Mohammad Sajjad Ali Khan, MBBS, FCPS (Medicine), PGT (Endo). Department of Medicine & Allied, Northwest General Hospital & Research Centre, Peshawar, Khyber Pakhtunkhwa, Pakistan; 4 Dr. Najmush Shakireen, MBBS. Department of Medicine & Allied, Northwest General Hospital & Research Centre, Peshawar, Khyber Pakhtunkhwa, Pakistan; 5 Dr. Iftikhar Ali, PharmD, MPhil, MPH. Pharmacy Unit, Paraplegic Centre, Hayatabad, Peshawar, Pakistan

**Keywords:** Urinary tract infections, Diabetes mellitus, Antibiotics sensitivity, Glycemic control

## Abstract

**Objective:**

To determine causative uropathogens and their antibiotic susceptibility pattern among Type-2 diabetics (T2D) with good and suboptimal glycemic control.

**Methods:**

A hospital based cross-sectional study was carried out in Peshawar from April–October, 2019. Four hundred consecutive T2D patients with symptomatic UTI or showing numerous pus cells on routine urinary examination attending outpatient clinic were included. As per the guidelines of the Clinical and Laboratory Standards Institute (CLSI), the urine samples collected were checked for identification of uropathogen by culture. Disc diffusion method was used to determined antimicrobial susceptibility.

**Results:**

Of the total (n=400) T2D patients, 205 (51.25%) showed microbial growth. Mean age of patients with UTI was 63.26 ±12.30 years. About two-third (63.9%) of the patients were females. Mean HbA1c was 8.80±2.20%. The frequency of patients with UTI was noticeably greater in the suboptimal glycemic control group 178(86.3%) compared to good control glycemic patients 27(13.7%). Significant mean difference in glycemic levels were observed (HbA1c = 5.86±0.48 and HbA1c = 9.25±2.02, respectively, *P* < 0.001). E. coli was the predominant pathogen isolated 120(71%), followed by Klebsiella pneumonia Spp (K. pn) 35(17.1%), Pseudomonas auregonosa (P. aeruginosa) 14(6.83%), Enterococcus 12 (5.85%) and Candida Spp were 2(0.98%). Both gram positive and negative-bacteria were highly susceptible to imipenem, meropenem, fosfomycin and nitrofurantoin.

**Conclusion:**

The frequency of UTI in diabetics was higher in female in comparison to male, and was significantly greater in the suboptimal glycemic control group. E. coli was the most typical isolate followed by K. pn. Imipenem, meropenem, fosfomycin and nitrofurantoin had high susceptibility profile against the isolated pathogens.

## INTRODUCTION

Diabetes Mellitus (DM) is a significant public health issue worldwide and has emerged as a significant socio-economic burden for developing nations.^1^ In 2017, worldwide 451 million people were diabetics, and by 2045 the number is expected to exceed by 693 million figures. According to latest Pakistan national diabetes survey about 26.3% of the local population age over 19 is diabetic.^2^

Urinary tract infection (UTI) is among the most common medical condition seen in all age groups with DM. Diabetic patients are highly susceptible to UTI compared to non-diabetics.^3^ Severe UTI and associated complications can be a significant cause of morbidity and mortality. Moreover, UTI is related to higher medical cost associated with its management. Possible mechanism of the UTI in DM might be neuropathy caused by hyperglycemia resulting in neurogenic bladder, urinary stasis and increasing probability of infection.^4^ Other possible explanation could be, decreased neutrophil activity, decrease urinary cytokines, and leukocyte concentrations, which could promote the adhesion of microorganisms to uroepithelial cells.^3^,^5^ Furthermore; hyperglycemia promotes the colonization and growth of a range of organism.^6^ Literature support that E. coli is the most common isolate followed by klebsiella pneumoniae, enterococci, pseudomonas, citrobacter, serratia, gram positive cocci, proteus and candida.^3^,^4^,^6^

Strict glycemic control in DM may help in decreasing the incidence of UTI, further the periodic screening and identification of the causative agent and proper management according to susceptibility pattern may decrease the associated complications and mortality.^3^,^5^

The emergence of UTIs caused by drug resistant strains is mounting both in community and healthcare setups and the situation is challenging in country like Pakistan due to irrational use of antibiotics.^7^ Moreover, very little information is available regarding the microbial etiology and their antimicrobial resistance pattern in diabetic patients with UTI in Pakistan in respect to glycemic status. Thus, this study was designed to find out the causative uropathogens and their antibiotic susceptibility pattern among patients with T2D with good and suboptimal glycemic control.

## METHODS

A prospective observational study was performed in Northwest General Hospital and Research Centre, Peshawar [April–October, 2019]. A total of (n=400) T2D patients with disease duration >1 year having symptomatic UTI (These symptoms include: frequency, urgency, dysuria, and suprapubic pain for lower UTI; and costovertebral angle pain/tenderness, fever, and chills, with or without lower urinary tract symptoms for upper UTI) or showing numerous pus cells on urine routine examination, attending outpatients clinic was considered for the study. The anticipated sample size for the study was determined using the formula: n = z^2^ pq/d^2^. The frequency of 52.7% reported in a study^7^ carried out in DM population in Karachi was used.

Of the total, n=205 patients had culture proven UTI, which were further analysed for socio-demographic and clinical characteristics, causative organism of UTI, glycemic status and antimicrobial susceptibility. Sterile urine samples and contaminated or those samples which showed mixed growth of microorganisms, likely due to improper handling were excluded. Those patients who were using Sodium-glucose co-transporter-2 (SGLT-2) or had received antibiotics within the last 72 hours of their presentation were also excluded.

Data regarding clinico-demographic profile were recorded using structured format. HbA1c was used as a glycemic control index. HbA1c levels more than 6.5% were considered suboptimal controlled diabetes. Hospitals ethics and review committee approved the study protocol [Ref. No: NwGH/5423].

The urine sample was collected in a sterile container. Sample was then inoculated on cled (cystine, lactose, electrolyte deficient) media and incubated at 37^o^ for 24-48 hours. After growth, organisms were classified by standard protocols using various identification and biochemical tests i.e. colony morphology, gram staining, positive oxidase reaction, production of pyocyanin on Mueller-Hinton agar (Oxide, Ltd, UK), citrate utilization and growth at 42^o^.

Antibiotics susceptibility was tested by Kirby-bauer’s disc diffusion method^8^ whereas sensitive and resistive organisms were labelled as per CLSI guidelines after determining zone of inhibition. The antibiotics disc that were used to classify the susceptibility pattern of bacterial pathogen include sulfamethoxazole/Trimethoprim (TMP/SMX) (25mcg), amoxiclave/Culvunate (30mcg), fosfomycin (50mcg), ciprofloxacin (5mcg), piperacillin / tazobactam(110μg), cefotaxime (30mcg), amikacin (30mcg) meropenem (10mcg), ceftazidime (30mcg), imipenem (10mcg), cefoperazone-sulbactam (30mcg), nitrofurantoin (300mcg) and cefipime (30mcg). With these antibiotics microorganism were marked as susceptible or resistant. In this study intermediate susceptibility was labelled as susceptible. A different phenomenon produced by microorganisms identified by modified double disc synergy test such as extended spectrum beta-lactamases (ESBL), cephalosporinase etc were also recorded.

Data analysis was done using SPSS version 20®. Number and proportions were calculated for categorical data and mean [SD] was calculated for continuous data. Statistical significance was determined using Chi Square statistics for categorical data or t- test for numerical data between the groups. Statistical significance was set at < 0.05.

## RESULTS

Mean age of patients was 63.26 ±12.30 years. The age ranges between 25-92 years, while majority 69.3% were in the age category of 51-75 years. Among the total patients with UTI, 63.90% were female patients. Culture confirmed UTI was 51.25%. Majority 66(32.20%) of the patients had DM >10 years [(Duration of DM (groups)] 11-15 years. Average comorbid conditions were 1.71±0.86. Most of the patients 139(67.80%) were hypertensive. About 38% patients had CKD. Mean HbA1c level was 8.80±2.20%.

Nearly half (49%) of the patients had previous history of culture proven UTI, and similarly the treatment modality of DM was insulin in 48.78%, (n=100) Table-I. The patients were classified corresponding to the glycemic status; patients with good glycemic control (HbA1c ≤6.5) and those with suboptimal control (HbA1c > 6.5) and their cross tabulation is depicted in Table-I. The number of patients in the suboptimal group, 86.8% was significantly higher compared with the good glycemic control group (13.2%).

**Table-I:** Background and clinical characteristics of the T2D patients and cross tabulation based on the glycemiac status.

Characteristic	N (%)	Good control HbA1c≤6.5 N=27	Suboptimal control HbA1c>6.5 N=178	P-value
Age (year) Mean(SD)	63.26±12.30	67.00±13.03	62.69±12.12	0.090
*Gender*				1.000
Male	131(63.90)	17(63.0)	114(64.0)	
Female	74(36.10)	10(37.0)	64(36.0)	
Co-morbid conditions	1.71±0.86	1.760±0.925	1.705±0.842	0.769
*Hypertension*				
Yes	139(67.80)	20(76.9)	119(69.2)	0.497
No	59(32.20)	6(23.1)	53(30.8)	
*Chronic kidney disease*				
Yes	78(38.05)	10(38.5)	68(39.5)	1.000
No	120(61.95)	16(61.5)	104(60.5)	
*Ischemic heart disease*				
Yes	34(16.6)	03(11.5)	31(18.0)	0.580
No	164(83.4)	23(88.5)	141(82.0)	
*Chronic liver disease*				
Yes	06(2.9)	1(3.7)	5(2.8)	0.576
No	192(97.1)	26(96.3)	173(97.2)	
*Hypothyroidism*				
Yes	5(2.4)	0	5(2,8)	0.490
No	193(97.6)	27(100)	173(97.2)	
*Cerebrovascular accident*				
Yes	20(9.8)	2(7.4)	18(10.1)	0.492
No	178(90.2)	25(92.6)	160(89.9)	
*Duration of diabetes (years)*				
<5	35(17.1)	6(23.1)	29(16.9)	0.649
5-10	61(29.8)	5(19.2)	56(32.6)	
11-15	66(32.2)	8(30.8)	54(31.4)	
16-20	31(15.2)	5(19.2)	23(13.4)	
>20	12(5.9)	2(7.7)	10(5.8)	
HbA1c (%) Mean(SD)	8.80±2.20	5.86±0.482	9.25±2.022	<0.001
*Past history of UTI*				
Yes	101(48.78)	16(59.2)	85(47.8)	0.293
No	103(51.22)	11(40.7)	93(52.2)	
*Treatment modality*				
OHA	105(51.22)	15(57.7)	90(51.0)	0.531
Insulin	100(48.78)	11(42.3)	88(49.0)	

The mean age was lower for the patients with suboptimal control diabetes (62.69±12.12) when compared to that (67.00±13.03 years) for the patients with good glycemic control (*P*>0.05). In both groups considerable mean difference in glycemic levels were observed (HbA1c = 5.86±0.48 and HbA1c = 9.25±2.02, respectively, *P*<0.001). Females showed much higher prevalence of UTI than males. The proportion of female patients was higher than male patients as 63.90% of UTIs of the total patient were in females as compared to 36.10% in males. The trend of gender distribution was comparable in both patient groups [HbA1c =≤6.5, and HbA1c= >6.5]. However, no statistically significant association was found in gender, comorbid conditions, duration of DM, previous history of UTI and the treatment modality between the two groups.

Compared to good glycemic control, in the patients with suboptimal control, a distinct rise of cases was observed, patients with ages more than 50, had high frequency of UTI (males 49, females 93). This same pattern has also been noted for both males and females, given the low number of males with UTI in this study, especially in the control glycemic group.

The occurrence and the distribution of microorganisms isolated from urine (Bacteria and yeast) are depicted in Table-II according to gender for both patients groups. These isolates in both gender characterised clinically important pathogens. E. coli was the predominant pathogen isolated from urine samples in both females and males, as well as from both patient groups.

**Table-II:** Isolated Uropathogen causing UTI among DM patients.

Microorganism		Glycemic status			

		HbA1c≤6.5		HbA1c>6.5	

Gram Negative	Total	Female	Male	Female	Male
E.coli ESBL	86	8	2	50	26
E.coli cephalosporinase producer	17	2	1	11	3
proteus mirabilis	2	1	0	0	1
Klebsiella pneumoniae ESBL	11	0	0	8	3
Klebsiella cephalosporinase producer	10	0	0	8	2
Klebsiella carbapenemase producer	7	0	0	5	2
Pseudomonas auregonosa	14	2	3	3	6
E.coli	17	2	0	10	5
Klebsiella pneumonia	7	0	1	3	3
Serratia marcescens	2	0	0	0	2
Providencia stuartii	3	1	0	2	0
Enterobacter cephalosporiase producer	1	0	0	0	1
Providencia cepholosorinase producer	2	0	1	0	1
Proteus vulgaris ESBL	1	0	0	1	0
Serratia Amp-C producer	1	0	0	0	1
Providencia ESBL	1	0	1	0	0
*Gram positive*					
Staphylococcus epidermidis	6	0	1	4	1
Staphylococcus aureus	3	0	0	2	1
Enterococus faecalis	12	1	0	5	6
*Yeast*					
Candida species	2	0	0	2	0

Total	205	17	10	114	64

Of the E.coli (ESBL) isolates (n=86), 97.6% were susceptible to imipenem, 95.3% to meropenem and 90 to fosfomycin. Susceptibility of E.coli (ESBL) to fosfomycin was 82%. The percent susceptibility of E.coli (cephalosporinase) isolates (n=17) were noted to be 100, 94, and 70 to imipenem, meropenem and nitrofurantoin respectively. A total of 11 isolates of k. pn (ESBL) showed 70% susceptibility to fosfomycin, 66.7 to amikacin, 100 to meropenem and imipenem while 60 to nitrofurantoin. Of a total of 6 isolates tested, 100% k.pn (carbapenamanase) showed sensitivity to fosfomycin, whereas the susceptibility to nitrofurantion, imipenem and meropenem were reported to be 57%, 43% and 28% respectively the details can be seen in Table-III.

**Table-III:** Drug Susceptibility of predominant isolated microorganism.

Growth	Pattern	E.coli [ESBL]%	E.coli [ESC-producing]%	K. pn [ESBL]%	KPC producing%	P. auregonosa%	E.coli%	K. pn%	Enterococus%
Trimethoprim-Sulfamethoxazole	S	22.8	5.9	20.0	20.0	66.7	43.8	71.4	0.0
	R	77.2	94.1	80.0	80.0	33.3	56.3	28.6	100.0
Amoxicillin-clavulanic acid	S	2.5	0.0	18.2	16.7	66.7	52.9	71.4	41.7
	R	97.5	100.0	81.8	83.3	33.3	47.1	28.6	58.3
Fosfomycin	S	90.7	82.4	72.7	100.0	80.0	100.0	100.0	85.7
	R	9.3	17.6	27.3	0.0	20.0	0.0	0.0	14.3
Ciprofloxacin	S	9.9	0.0	36.4	28.6	76.9	52.9	71.4	0.0
	R	90.1	100.0	63.6	71.4	23.1	47.1	28.6	100.0
Piperacillin-Tazobactam	S	0.0	0.0	25.0	33.3	90.0	50.0	100.0	0.0
	R	100.0	100.0	75.0	66.7	10.0	50.0	0.0	100.0
Amikacin	S	70.2	30.0	66.7	50.0	50.0	100.0	75.0	20.0
	R	29.8	70.0	33.3	50.0	50.0	0.0	25.0	80.0
Meropenem	S	95.3	94.1	100.0	28.6	78.6	82.4	85.7	75.0
	R	4.7	5.9	0.0	71.4	21.4	17.6	14.3	25.0
Ceftazidime	S	4.3	0.0	12.5	16.7	72.7	37.5	60.0	0.0
	R	95.7	100.0	87.5	83.3	27.3	62.5	40.0	100.0
Imipenem	S	97.6	100.0	100.0	42.9	78.6	82.4	85.7	75.0
	R	2.4	0.0	0.0	57.1	21.4	17.6	14.3	25.0
Cefoperazone-sulbactam	S	0.0	0.0	12.5	33.3	71.4	56.3	60.0	20.0
	R	100.0	100.0	87.5	66.7	28.6	43.8	40.0	80.0
Nitrofurantoin	S	82.6	70.6	60.0	57.1	83.3	88.2	85.7	50.0
	R	17.4	29.4	40.0	42.9	16.7	11.8	14.3	50.0
Cefipime	S	14.5	11.8	30.0	16.7	33.3	40.0	83.3	20.0
	R	85.5	88.2	70.0	83.3	66.7	60.0	16.7	80.0

*Abbreviations:* R; Resistance, S; Sensitive, ESBL; Extended spectrum beta-lactamase, ESC; Extended spectrum cephalosporinase, KPC; Klebsiella pneumoniae carbapenemase, K. pn; Klebsiella pneumoniae

## DISCUSSION

This study results showed that significant number of patients had culture proven UTI. This frequency is comparable to the results of the locally and regionally conducted recent studies which observed a frequency of 50.7^5^ and 52.76%^3^ respectively. It is relatively high as compared to other studies which reported 13.8, 34.5, 34, 35, and 43% respectively.^9^-^12^ This may be due to the fact that majority 29.4% and 32.20% of the study patients were diabetic for long period 5-10 and 11-15 years respectively. History of diabetes and the occurrence of long-term complications in these people are important possible causes for UTI, instead of just current glucose control.^3^,^13^ High UTI incidence among these patients may be attributed to diminished antibacterial activity, neutrophil activity defects, protein abundance and increased adhesion to uroepithelial cells.^14^,^15^ Other worth mentioning reason can be the geographic variation, poor personal hygiene practices, ethnicity and health education.^5^,^7^

Moreover, in this study 178(86.83%) of the UTI cases were found in patients with uncontrolled diabetes, consistent with previous observation of this phenomenon.^3^,^5^ In this study, about two third 63.90 of the DM patients with UTI were females. A number of reports revealed the predominance of UTI in women as compared to men.^3^,^16^ Furthermore, such trend of gender ratio was quite close for both diabetic groups with managed and uncontrolled glycaemia, showing that glycemic regulation has no effect on the distribution of UTI by sex.

Different results have been documented in earlier studies where there were no major variations in the frequency of bacteriuria for both males and females.^3^,^17^ In comparison, Geerlings et al. indicated that bacteriuria was more common in women with uncontrolled diabetes as compared to non-diabetic women.^13^ Since most earlier studies of UTI in diabetics have been performed in females, there is conflicting evidence of aspects of UTI in diabetic males,^18^ and this data represents the first in Pakistan to compare the impact of glycemic regulation on UTIs in diabetics.

In our study patients were relatively older as compared to other studies.^3^,^5^,^12^,^17^ Nearly 70% patients with UTI were in the age range of 51-75 in this study. Zubair et al, reported that most of their patients with UTI were between the ages of 51-60 years^7^ while a study by Kumar et al, reported the ages between 31-40 years.^19^ The reason for this variation may be due to late diagnosis of diabetes, ethnic variation and attitude of general population for seeking medical attention. On the contrary, old age has been generally recognized as a contributing factor for people with T2D.^3^,^7^

In this study compared to strict glycemic control group, those with suboptimal control a distinct rise of UTI cases with age was observed as 70.18% of cases were noticed in female with age groups of 51-75 years. Almost same pattern has also been noted in both males and females, given the low proportion of men with UTI in this analysis, notably in the controlled glycemic group. Similar results have been described by Sewify et al, where irrespective of the sex and smaller number of patients in the controlled group an almost comparable trend of the cases was noted through all age ranges.^3^ In this study, the number of patients was noticeably greater in the suboptimal control group (86.3%) in comparison to the good control group (13.7%).

Keeping in mind this small number of patients in controlled group, which is insufficient to explain this disparity in age patterns while understanding that the incidence of T2D and its complications is increases with age. It should be noted that the majority of people enrolled in our study had DM for more than 10 years.

E.coli (ESBL) was among the most common gram negative isolate followed by Pseudomonas auregonosa, Klebsiella pneumoniae (ESBL) and Klebsiella pneumonia cephalosporinase producers. These isolates were equally present in both groups. Both, E. coli and K. pneumoniae strains were the most common uropathogens. Similar observations were earlier reported and established the prevalence of ESBL-producing E. coli and K.pn species in diabetics and in non-diabetic subjects.^6^,^16^,^17^ These findings together may indicate a direct relationship between glycemic control and UTI with ESBL-producing strains.

Among the gram positive, Enterococus represented 5.9% of the isolated pathogens, whereas staph epidermidis was found in three of the cases and only about 1% of cases were assigned to candida species. Enterococcus Spp 0.93, Candida 5.61 was found in a local study and 18.4%, 8% from other similar studies, correspondingly.^3^,^7^,^12^

Gram-negative bacteria exhibited an alarming resistance to first and second line agent’s trimethoprim-sulphamethoxazole, cephalosporins, amikacin, amoxicillin/clavulanic acid and flourquinolones in this study. Our study indicates that these antibiotics cannot be used as an empirical treatment for UTI in DM patients due to the emergence of increasing antimicrobial resistance to first and second line drugs. Therefore, emerging resistance to these antibiotics in diabetics may be considered while developing new guidelines. Although, Gram-negative isolates were sensitive to carbapenems and antipseudomonal penicillins, these drugs cannot be recommended in outpatient settings due to their supervised drug administration protocols. Also, where fosfomycin and nitrofurantoin have shown intermediate to full susceptibility pattern, more than 50% of the isolated Gram-positive isolates were resistant to these antibiotics. This result questions the effectiveness of fluoroquinolone for the empiric treatment. Though, all the isolated uropathogens were susceptible to nitrofurantoin. This is consistent with earlier studies.^20^,^21^ Therefore, nitrofurantoin can be used as the drug of choice in the study area as a potential treatment of UTI.

### Limitations of the study

The worth mentioning limitation of the study is not taking into account the history of nephrolithiasis or any bladder disorders. Other limitations include the sampling technique and study design while intermediate was considered susceptible. It was not determined if the diabetes control improved, would chances of UTI reduce or not and should be studied prospectively. The proposed study would generate more useful data and has not been done in Pakistan.

## CONCLUSION

High frequency of UTI in diabetic patients was noted. Female showed high frequency of UTI compared to male and the number of patients with UTI was noticeably higher in the suboptimal group. E. coli species and k.pn species were the most common isolates. Imipenem, meropenem, fosfomycin and nitrofurantoin had high susceptibility profile against the isolated pathogens. Such findings illustrate the significance of glycemic regulation in diabetics in reducing UTI regardless of age and sex. Monitoring of isolated microorganisms’ antimicrobial susceptibility patterns allows appropriate use of antimicrobial agents for the management of UTIs, preventing the development of antibiotic-resistant urinary organisms.

### Authors’ contributions:

**SA and IA:** Conception & design.

**SA:** Data collection.

**AI:** Data analysis and interpretation/results.

**AI, AH and MSAK:** Manuscript drafting and writing.

**AH, SA and NUS:** Language editing/appropriateness, critical revision.

All authors read and approved the final version of the paper.

The principal investigator is responsible and accountable for the accuracy or integrity of the work.

## References

[1] Ijaz M, Ali I, Hussain A (2020). Diabetes mellitus in Pakistan:the past, present, &future. Int J Diabetes Developing Countries.

[2] Basit A, Fawwad A, Qureshi H, Shera A (2018). Prevalence of diabetes, pre-diabetes and associated risk factors:second National Diabetes Survey of Pakistan (NDSP) 2016-2017. BMJ Open.

[3] Sewify M, Nair S, Warsame S, Murad M, Alhubail A, Behbehani K (2016). Prevalence of urinary tract infection and antimicrobial susceptibility among diabetic patients with controlled and uncontrolled glycemia in Kuwait. J Diabetes Res.

[4] Nitzan O, Elias M, Chazan B, Saliba W (2015). Urinary tract infections in patients with Type-2 diabetes mellitus:review of prevalence, diagnosis, and management. Diabetes Metab Syndr Obes.

[5] Bashir H, Saeed K, Jawad M (2017). Causative agents of urinary tract infection in diabetic patients and their pattern of antibiotic susceptibility. Khyber Med Univ J.

[6] Hamdan HZ, Kubbara E, Adam AM, Hassan OS, Suliman SO, Adam I (2015). Urinary tract infections and antimicrobial sensitivity among diabetic patients at Khartoum, Sudan. Ann Clin Microbiol Antimicrob.

[7] Zubair KU, Shah AH, Fawwad A, Sabir R, Butt A (2019). Frequency of urinary tract infection and antibiotic sensitivity of uropathogens in patients with diabetes. Pak J Med Sci.

[8] Clinical and Laboratory Standards Institute (CLSI) (2015). Document M100-S25:Performance Standards for Antimicrobial Susceptibility Testing:Wayne, PA, USA.

[9] Nigussie D, Amsalu A (2017). Prevalence of uropathogen and their antibiotic resistance pattern among diabetic patients. Turk J Urol.

[10] Sarvepalli VK, Patak NP, Kandati J, Pathapati RM, Buchineni M (2015). Prevalence of uropathogens and their antibiogram in diabetic patients a cross sectional study. Int J Curr Microbiol App Sci.

[11] Sharma V, Gupta V, Mittal M (2012). Prevalence of uropathogens in diabetic patients and their antimicrobial susceptibility pattern. Natl J lab Med.

[12] Acharya D, Bogati B, Shrestha G, Gyawali P (2015). Diabetes mellitus and urinary tract infection:spectrum of uropathogens and their antibiotic sensitivity. J Manmohan Mem Inst Health Sci.

[13] Geerlings SE, Stolk RP, Camps M, Netten PM, Collet TJ, Hoepelman A (2000). Risk factors for symptomatic urinary tract infection in women with diabetes. Diabetes care.

[14] Gutema T, Weldegebreal F, Marami D, Teklemariam Z (2018). Prevalence, Antimicrobial Susceptibility Pattern, and Associated Factors of Urinary Tract Infections among Adult Diabetic Patients at Metu Karl Heinz Referral Hospital, Southwest Ethiopia. Int J Microbiol.

[15] Cheesbrough M (2006). District laboratory practice in tropical Countries Part 2,:Cambridge University Press, New York, NY, USA.

[16] Al Benwan K, Al Sweih N, Rotimi VO (2010). Etiology and antibiotic susceptibility patterns of community-and hospital-acquired urinary tract infections in a general hospital in Kuwait. Med Princ Pract.

[17] Aswani SM, Chandrashekar U, Shivashankara K, Pruthvi B (2014). Clinical profile of urinary tract infections in diabetics and non-diabetics. Australas Med J.

[18] Nicolle LE (2014). Urinary tract infections in special populations:diabetes, renal transplant, HIV infection, and spinal cord injury. Infect Dis Clin N Am.

[19] Kumar Jha P, Baral R, Khanal B (2014). Prevalence of uropathogens in diabetic patients and their susceptibility pattern at a tertiary care center in Nepal-a retrospective study. Int J Bio Lab Sci.

[20] Alebiosu C, Osinupebi O, Olajubu F (2003). Significant asymptomatic bacteriuria among Nigerian Type-2 diabetics. Natl Med Assoc.

[21] Kabew G, Abebe T, Miheret A (2013). A retrospective study on prevalence and antimicrobial susceptibility patterns of bacterial isolates from urinary tract infections in Tikur Anbessa Specialized Teaching Hospital Addis Ababa, Ethiopia 2011. Ethiop J Health Dev.

